# A Report on the Hygiene of the United States Army

**Published:** 1875-09

**Authors:** 


*A Report on the Hygiene of the United States Army, with Descriptions of the Military Posts.* Circular No. 8, War Department, Surgeon-General's Office. Washington: Government Printing Office, 1875.

The report on the Hygiene of the Army is from the pen of Assistant-Surgeon J. S. Billings, and occupies the first fifty-five pages of the report. This report shows an advance in the hygienic regulations of the army during the last five years which reflects credit upon the Medical Department, The effort is being made to place everything pertaining to tho soldiers' wants, as clothing, food, etc., of the highest standard of excellence.

The discussion of hospital construction is an interesting one Dr. Billings accepts the plan of the barrack hospital of wood intended for temporary occupancy as the best yet advanced. He does not think that the source of contagion may be looked for as much in the saturation of the walls of the building as in the furniture, clothing, etc.

The greater portion of the work is filled with a description of the military posts throughout the United States.

